# Aeromonas hydrophila Bacteremia in an Immunocompromised Patient

**DOI:** 10.7759/cureus.111835

**Published:** 2026-06-30

**Authors:** Rhianna M Haynes, Joshua Can

**Affiliations:** 1 Internal Medicine, Saba University School of Medicine, The Bottom, NLD

**Keywords:** aeromonas hydrophila, clinical case report, gram-negative bacteremia, immunocompromised patient, kidney transplant recipient

## Abstract

*Aeromonas hydrophila *(*A. hydrophila*)is a facultative anaerobic, Gram-negative, water-dwelling bacterium that can cause a wide range of infections, including bacteremia, especially in immunocompromised patients. This report presents the case of a 68-year-old female patient with a history of renal transplant and chronic immunosuppression, who developed bacteremia caused by *A. hydrophila *after being exposed to raw crab. Initial presentation included an acute kidney injury and gastrointestinal symptoms, but she had an abnormal clinical course, as she did not have a fever or leukocytosis. Empiric piperacillin-tazobactam was initiated, then later de-escalated to intravenous (IV) ceftriaxone and oral doxycycline following susceptibility testing. This treatment led to a swift recovery. The case presentation emphasizes the importance of having a high index of suspicion in immunocompromised patients, as they may present atypically. A thorough history, including exposure history, is vital for proper identification of rare pathogens such as* A. hydrophila. *Appropriate treatment following diagnosis is vital to reduce morbidity and mortality associated with* Aeromonas *bacteremia.

## Introduction

*Aeromonas hydrophila* (*A. hydrophila*) is a facultative anaerobic, Gram-negative, rod-shaped, water-dwelling bacterium that has been known to cause infections both locally and systemically [1]. Immunocompromised individuals are most commonly affected by* A. hydrophila*, predominantly by bacteremia, gastrointestinal, or soft-tissue infections [2]. These infections and tissue injury are mediated by virulence factors such as cytotoxins, enterotoxins, and hemolysins [1,3]. Although *Aeromonas *bacteremia is uncommon, it carries substantial morbidity and mortality [2,4].

This report describes a patient with bacteremia due to pansensitive *A. hydrophila* who demonstrated rapid clinical improvement following treatment. The patient, who provided a reliable history, was found to have likely acquired the infection through exposure to raw crabs in the setting of ongoing immunosuppression following a prior renal transplant. The patient had an atypical presentation of bacteremia. We present this case to aid in the appropriate recognition and treatment of *Aeromonas *bacteremia in immunocompromised patients, based on current literature and clinical understanding.

## Case presentation

A 68-year-old female patient from southern Louisiana with a past medical history of autosomal dominant polycystic kidney disease with resulting right nephrectomy and renal transplant in 2017 on immunosuppressants tacrolimus and mycophenolate presented to the emergency department as a referral from urgent care due to dizziness, hypotension, fever, vomiting, and non-bloody, non-foul-smelling diarrhea for the past six days. The patient reported all symptoms resolving two days prior to arrival, except for persistent diarrhea. Vital signs, an anteroposterior (AP) portable chest X-ray, and a 12-lead EKG were within normal limits. Serum samples exhibited hypokalemia, elevated blood urea nitrogen (BUN), elevated creatinine, and decreased glomerular filtration rate (GFR) (Table 1). An acute kidney injury (AKI) was present due to dehydration secondary to diarrhea, and the patient reported decreased oral intake after feeling unwell. The patient was treated with oral potassium, resulting in an increase in potassium level, and intravenous (IV) fluids, which elicited a slight decrease in creatinine and BUN (Table 1). The patient then requested discharge and was sent home with instructions to follow up with nephrology. Urinalysis performed a day later showed hazy color, trace protein, trace ketones, leukocytosis (1+), and many bacteria, along with two blood cultures positive for Gram-negative rods, revealing infection. The patient was notified by telephone.

**Table 1:** Patient laboratory values before and after the treatment

Labs	Pre-treatment	Post potassium and fluids	Post the initiation of Zosyn	Baseline
Potassium	2.9 mmol/L	3.4 mmol/L	3.1 mmol/L	3.5-5 mmol/L
Blood urea nitrogen (BUN)	35 mg/dL	31 mg/dL	21 mg/dL	14 mg/dL
Creatinine	1.6 mg/dL	1.5 mg/dL	1.1 mg/dL	0.9 mg/dL
Glomerular filtration rate (GFR)	25 ml/min/1.73 m²	38 mL/min/1.73 m²	55 mL/min/1.73 m²	>60 mL/min/1.73 m²

Upon returning, the patient started feeling weak. She was now hypertensive (149/60 mm Hg) and tachycardic (124 bpm), but still afebrile with no leukocytosis or urinary symptoms. A second set of blood cultures was collected, and piperacillin-tazobactam (Zosyn) 4.5 g in 5% dextrose in water 100 mL via IV piggyback was started. The patient had a history of urinary tract infections, with the most recent occurring a year prior, during which *Klebsiella *species was identified. No recent infections or antibiotic use were noted. After starting Zosyn and receiving additional IV fluids on admission, BUN decreased, creatinine decreased, and GFR increased (Table 1), demonstrating improvement in AKI. On the second day of admission, the patient remained afebrile, but the review of systems was positive for chills, diarrhea, and dizziness. A third set of blood cultures was obtained. Two doses of Zosyn were given before switching to Ceftriaxone (Rocephin) 2 g every 24 hours.

On day three, blood cultures were positive for Gram-negative rods presumptively identified as A. hydrophila, in both the initial and the second set of blood cultures. Antibiotic sensitivity testing exhibited pansensitivity (Table 2). The patient remained afebrile and was on day 2 of Rocephin. The third set of blood cultures had no growth at six hours. It was discovered that the patient contracted the infection from handling crabs residing in water containing A. hydrophila. No other family members contracted an infection. The Infectious Diseases team was consulted.

**Table 2:** Drug sensitivity test of Aeromonas hydrophila

Antibiotic	Minimum Inhibitory Concentration (MIC) Quantitative	Minimum Inhibitory Concentration (MIC) Qualitative
Amikacin	≤16	Sensitive
Cefepime	≤2	Sensitive
Ceftriaxone	≤1	Sensitive
Genamicin	≤2	Sensitive
Piperacillin/Tazobactam	≤8	Sensitive
Tetracycline	≤4	Sensitive
Tigecycline	≤2	Sensitive
Tobramycin	≤2	Sensitive
Trimethoprim/Sulfamethoxazole	≤0.5/9.5	Sensitive

On day four of admission, the patient was hemodynamically stable and asymptomatic except for residual diarrhea, which decreased in frequency. The patient was discharged with oral doxycycline 100 mg twice daily for five days, having received three days of IV antibiotics during her hospital stay, and was instructed to follow up with family medicine, cardiology, infectious disease, and nephrology following hospitalization for bacteremia. The patient was advised to be cautious when handling raw seafood, to boil crabs at home, even if they are already boiled at the store, and to properly cook all seafood thoroughly.

One week post discharge, the patient followed up with their primary care doctor. The patient had completed her antibiotic course three days prior and was asymptomatic, stating that the diarrhea had improved since completing the course. The cardiology follow-up the next day was also unremarkable. Since the patient had bacteremia, a transthoracic echocardiogram was scheduled for two days later, which revealed no changes from prior scans and a normal ejection fraction of 60%. Two weeks after discharge, the patient followed up with the infectious disease specialist, satisfied that the third set of blood cultures had remained with no growth five days after collection. Nephrology follow-up occurred 16 days post discharge, with complete recovery of the patient's renal function tests to baseline. All unremarkable follow-ups and an asymptomatic patient revealed a full recovery and patient satisfaction with treatment.

## Discussion

*A. hydrophila* is a facultatively anaerobic, oxidase-positive, Gram-negative bacillus widely distributed in freshwater and marine environments and frequently isolated from contaminated food sources, particularly seafood [5]. It is an opportunistic pathogen capable of causing a spectrum of disease ranging from self-limited gastroenteritis to potentially fatal infections, including bacteremia [3,5].

Bacteremia due to *Aeromonas *species most commonly occurs in immunocompromised individuals, including those with malignancy, chronic liver disease, diabetes mellitus, and solid organ transplants [3,6]. Transplant recipients are particularly susceptible due to chronic immunosuppression and impaired innate immune responses. Notably, clinical presentation in these patients may be atypical. In contrast to the classic presentation of fever and leukocytosis in bacteremia, our patient remained afebrile and leukopenic despite confirmed bacteremia, underscoring the potential for blunted inflammatory responses and the importance of acting on microbiologic data, even in clinically stable patients.

Environmental exposure plays a central role in pathogenesis. *Aeromonas *species are commonly acquired through ingestion of contaminated food or water or via explicit interaction with aquatic environments [4,5]. Handling raw seafood has been cited as a source of infection, particularly in immunocompromised hosts. In this case, exposure to raw crabs provides a likely source, emphasizing the importance of a detailed exposure history in patients with bacteremia of unclear origin.

*Aeromonas *bacteremia is associated with significant mortality, reported between 20% and 40%, particularly in high-risk populations or with delayed treatment [4,5]. The organism produces inducible β-lactamases, conferring resistance to penicillins, but typically remains susceptible to third-generation cephalosporins, fluoroquinolones, carbapenems, aminoglycosides, and tetracyclines [4]. In this case, early empiric therapy with piperacillin-tazobactam followed by de-escalation to ceftriaxone and oral doxycycline, guided by susceptibility testing, resulted in rapid clinical improvement and complete resolution of infection.

## Conclusions

This case report presents *A. hydrophila* as the source of bacteremia in a patient with a previous solid organ transplant. Importantly, this case highlights possible atypical presentations of bacteremia, as seen by this patient’s lack of fever and leukocytosis. This atypical presentation can impair a timely diagnosis if there is a low clinical suspicion. A critical piece in identifying this bacterial infection is a thorough exposure history, including contact with raw seafood. Empiric antibiotic therapy followed by targeted therapy, as guided by susceptibility testing, aids in improved clinical outcomes, including the high-risk, immunosuppressed patient presented in this case. Awareness of *A. hydrophila* and how it presents is crucial in identifying the source of infection and appropriate management, in turn, reducing morbidity and mortality. 
